# Lindsay & Blakiston’s Physician’s Visiting List, Diary, and Book of Engagements

**Published:** 1863-08

**Authors:** 


LINDSAY & BLAKISTON'S PHYSICIAN'S VISITING LIST, DIARY, AND BOOK OF ENGAGEMENTS. Thirteenth year of its publication.

The volume of this very convenient, we might say, almost indispensable, book, for 1864, is now ready, and offered to the profession at the following prices:—

| | | | | | |
|---|---|---|---|---|---|
| For | 25 | patients, weekly, | plain, | .............. | $ 63 |
| " | 25 | do. | do. | tucks, ............ | 1.00 |
| " | 50 | do. | do. | plain, .............. | 75 |
| " | 50 | do. | do. | tucks, ............ | 1.25 |
| " | 100 | do. | do. | do. ............ | 2.00 |

An *interleaved* edition is also published, for the use of such physicians as compound and furnish their own medicines to their patients; and sold at a small advance on the above prices.

